# Hydrogel Loaded with Extracellular Vesicles: An Emerging Strategy for Wound Healing

**DOI:** 10.3390/ph17070923

**Published:** 2024-07-10

**Authors:** Yucan Yang, Huizhi Chen, Yunjie Li, Junting Liang, Feng Huang, Liyan Wang, Huilai Miao, Himansu Sekhar Nanda, Jin Wu, Xinsheng Peng, Yubin Zhou

**Affiliations:** 1 Key Laboratory of Liver Injury Diagnosis and Repair, and Department of Hepatobiliary Surgery, The 2nd Affiliated Hospital of Guangdong Medical University, Zhanjiang 524001, China; volcano-fish@outlook.com (Y.Y.); chenhuizhimail@126.com (H.C.); yunjieli2000@163.com (Y.L.); liangjt2021@163.com (J.L.); huangfeng202202@163.com (F.H.); wly98gd@163.com (L.W.); miaohl-gdwk@gdmu.edu.cn (H.M.); 2 Guangdong Provincial Key Laboratory of Research and Development of Natural Drugs, Dongguan Key Laboratory of Advanced Drug Delivery and Biosensing Research and Development, School of Pharmacy, and Dongguan Innovation Institute, Guangdong Medical University, Dongguan 523808, China; 3 Biomaterials and Biomanufacturing Laboratory, Discipline of Mechanical Engineering, PDPM Indian Institute of Information Technology Design and Manufacturing, Jabalpur 482005, Madhya Pradesh, India; himansu@iiitdmj.ac.in; 4 State Key Laboratory of Optoelectronic Materials and Technologies and the Guangdong Province Key Laboratory of Display Material and Technology, School of Electronics and Information Technology, Sun Yat-sen University, Guangzhou 510275, China; wujin8@mail.sysu.edu.cn

**Keywords:** extracellular vesicles, hydrogels, wound healing, controlled release, tissue engineering

## Abstract

An increasing number of novel biomaterials have been applied in wound healing therapy. Creating beneficial environments and containing various bioactive molecules, hydrogel- and extracellular vesicle (EV)-based therapies have respectively emerged as effective approaches for wound healing. Moreover, the synergistic combination of these two components demonstrates more favorable outcomes in both chronic and acute wound healing. This review provides a comprehensive discussion and summary of the combined application of EVs and hydrogels to address the intricate scenario of wounds. The wound healing process and related biological mechanisms are outlined in the first section. Subsequently, the utilization of EV-loaded hydrogels during the wound healing process is evaluated and discussed. The moist environment created by hydrogels is conducive to wound tissue regeneration. Additionally, the continuous and controlled release of EVs from various origins could be achieved by hydrogel encapsulation. Finally, recent in vitro and in vivo studies reported on hydrogel dressings loaded with EVs are summarized and challenges and opportunities for the future clinical application of this therapeutic approach are outlined.

## 1. Introduction

**Figure 1:**
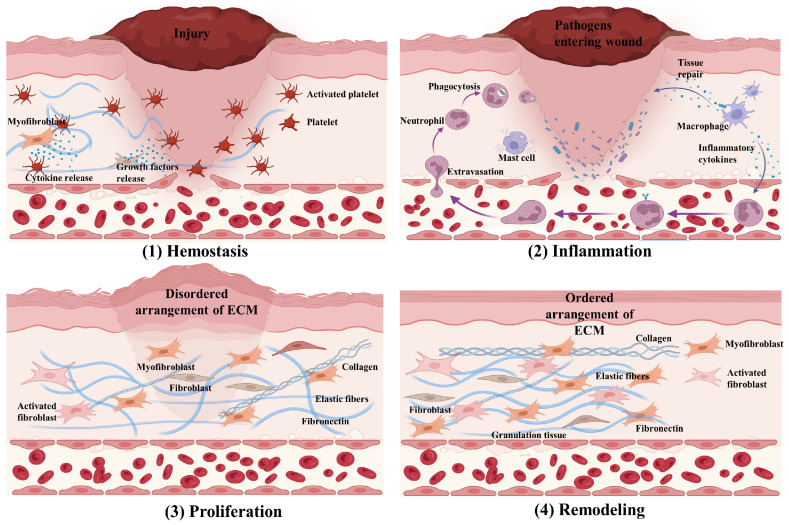
Wound healing process phase: (**1**) hemostasis; (**2**) inflammation; (**3**) proliferation; and (**4**) remodeling.

As the primary protective barrier of the human body, skin can withstand various external adverse factors such as harmful ultraviolet rays, mechanical wear, and parasites [1]. However, if skin damage occurs and results in structural and functional impairment, it disrupts the process of maintaining internal balance. Impaired wound healing is a significant health issue in our society that causes pain, suffering, psychological stress, and loss of quality of life for millions of people. It also puts tremendous pressure on medical systems and insurance companies, leading to increased costs. Rapid and effective wound closure is crucial for maintaining skin integrity and preventing infection. Wound healing is a complex process involving different events mediated by various cells and biological factors to repair injured tissues [2]. The process involves four sequential and overlapping stages: (1) hemostasis, (2) inflammation, (3) proliferation, and (4) maturation/remodeling, as shown in Figure 1. This requires the coordination of multiple cell types and molecular mechanisms that occur in a specific spatial and temporal manner. The correct initiation and orderly progression of these stages are critical for proper injury recovery [3].

## 2. Wound Healing

After skin damage, the first stage of wound healing is hemostasis. Blood vessels constrict to reduce blood flow and platelets aggregate to form a fibrin clot, preventing further bleeding and infection [4] and providing a temporary matrix for cell migration. Cytokines and chemokines released by platelets attract pro-inflammatory cells to the wound, which then enter the inflammatory phases [5]. Neutrophils enter the damaged tissue, releasing proteolytic enzymes, reactive oxygen species, and antimicrobial substances to eliminate bacteria and clear the damaged extracellular matrix (ECM) [6,7]. Inflammatory cytokines and growth factors attract and activate neutrophils and lymphocytes. After neutrophils clear pathogens, they undergo apoptosis, and macrophages phagocytize apoptotic cells and the damaged matrix. Macrophages play a key role in the inflammatory phase, polarizing to the M1 type early on, releasing a variety of pro-inflammatory factors [8]. After the early inflammatory phase subsides, neutrophils undergo senescence and apoptosis, and macrophages transform into the M2 type, secreting a variety of growth factors to promote cell proliferation and angiogenesis. In fact, the proliferative and remodeling phases of the wound occur simultaneously. At this time, a variety of cells proliferate and migrate to form granulation tissue to fill the damaged area [9]. The granulation tissue, composed of inflammatory cells, fibroblasts, and new capillaries, provides a new foundation for the migration of keratinocytes [10]. The expression of growth factors in the wound area increases, promoting the formation of new blood vessels, with vascular endothelial cells forming vascular sprouts and developing into microvessels [11]. Fibroblasts proliferate and synthesize at the wound edge, secreting type III collagen, fibronectin, hyaluronic acid, and other components to construct a new ECM, ultimately forming mature scar tissue. However, excessive matrix secretion may lead to the generation of scar tissue, which can impede the normal function of the skin. Based on this, it has been suggested that fibroblast cell-derived EVs can increase the expression of genes associated with scarless healing by increasing the expression of vascular endothelial growth factor (VEGF) and promoting the formation of skin appendages through the expression of β-catenin [12]. Keratinocytes interact with matrix metalloproteinases (MMPs) to break down and digest necrotic tissue fragments near the wound [13]. Other proteases are also involved in this process, degrading the temporarily loose granulation tissue at the wound edge to promote cell migration and proliferation, allowing the epidermis to stratify and regain functionality [14].

Traditional methods such as surgical debridement, the local/systemic application of antibiotics, and wound dressing are widely used in the clinical treatment of chronic trauma [15]. However, due to the intricate inflammatory milieu of diabetic wounds, the utilization of currently available materials is limited in promoting angiogenesis. Traditional wound dressings such as gauze, colloids, films, foam, and bandages serve specific functions like providing a physical barrier or removing exudate. However, they do not fulfill all the requirements for complete healing during the chronic wound healing process, where antimicrobial treatments are always required. Recently, inorganic nanomaterials such as silver, copper, zinc, and titanium, as well as natural ingredients, have been utilized and included in the design of antibacterial wound hydrogel dressings, additionally avoiding reliance on antibacterial drugs. However, it is essential to consider their potential toxicity and associated risks [16,17].

The direct transplantation of stem cells for tissue regeneration therapy has been extensively studied in recent years and has shown positive results [18,19]. However, the direct transplantation of exogenous stem cell therapy has limitations. The cells used in cell therapy are alive and must be applied to the patient very quickly. Once thawed, clinicians have limited time to apply them. Moreover, cell therapy may lead to complications, such as the secretion of proinflammatory molecules, nonspecific cell differentiation, and an immunoprotective response of transplanted stem cells [20,21]. Although cell-based therapies have been shown to be relatively safe, from a clinical perspective, cell-free therapies can avoid the negative effects of living cell therapies [16,22,23]. More importantly, an increasing amount of evidence has shown that the beneficial effects of stem cells primarily depend on their paracrine modes, such as growth factors, cytokines, and extracellular vesicles (EVs). In particular, EV-related pathways have attracted particular attention [24].

## 3. Extracellular Vesicles

EVs are phospholipid bilayer vesicles with a diameter ranging from 30 nm to 2000 nm that are actively secreted by cells [25]. They mediate cell–cell communication through cell membrane transfer and are capable of transferring lipid-coated signaling proteins and nucleotides between cells [26]. In 2008, it was reported that EVs can carry RNA and transfer between cells, representing a novel mechanism of bioactive molecular exchange [27]. As natural carriers of chemical drugs, compared with other artificial carriers (such as liposomes, microspheres, etc.), EVs contain a large number of membrane proteins on the surface, which can avoid phagocytosis by macrophages and prolong the half-life of drugs, which is highly beneficial to drug delivery [28,29]. EVs function as information carriers in multicellular environments. They serve as in vivo dynamic regulatory systems, where their content substances and functions can be adjusted based on their sources and the stimuli that promote their release [30]. Studies have shown that EVs from various origins can regulate cellular functions involved in wound healing, promote the growth of new blood vessels, stimulate collagen deposition, and reduce inflammation, thus expediting wound healing [31,32].

Although the mechanism of EVs acting as therapeutic protein carriers in the process of wound healing is not very clear, compared with traditional synthetic carriers, EVs still show positive pro-healing effects in wound healing [33], and the efficiency of loading and delivering active drugs to target cells is improved [34]. This provides a novel strategy for modern drug delivery. However, the commonly used method of EV administration is injection, which may induce secondary damage to the skin and result in the loss and waste of EVs. The fast clearance rate also affects the function of EVs [35]. In the absence of an effective carrier, maintaining a stable and effective concentration of EVs at the wound site for an extended period is challenging. Enzymes present at the wound site will also degrade EVs, thereby affecting the biological activity of EVs. Integrating them with novel biomaterials may be essential for avoiding fast clearance to advance tissue repair. Due to their inherent physical and chemical properties, such as sustained release and controlled release abilities and anti-infection properties, new biomaterials have exhibited superior performance [36]. In the process of wound repair, many types of biomaterials have been utilized, including hydrogels, electrospinning, hemostatic sponge scaffolds, and 3D-printed scaffolds. These materials possess the ability to regulate the wound process, promote collagen synthesis, direct cell migration, and facilitate angiogenesis to accelerate wound healing. Hydrogels have attracted extensive attention in bioengineering due to their ease of fabrication and ability to retain large amounts of water.

## 4. Hydrogels

Due to their simple physical isolation and moisture retention ability, hydrogels are employed widely in biological applications. However, to meet clinical needs and various demands, increasingly innovative and multifunctional hydrogels have been developed, such as antibacterial [37], anti-inflammatory, anti-oxidation, temperature-sensitive, pH-response, and drug delivery hydrogels [38]. Following the cross-linking of hydrophilic macromolecules, a three-dimensional network structure is formed inside the hydrogel. This three-dimensional network structure provides a device for the loading and sustained release of active ingredients such as EVs [39,40]. The pore size of the network in the hydrogel depends on the type of material, the concentration of the cross-linking agent, and the temperature and pH of the environment [41]. The ability of hydrogels to retain water and the sustained release of encapsulated therapeutic drugs are mainly determined by the pore size of the internal 3D network [42,43]. In general, it is easier to load EVs onto hydrogels with larger pore sizes, but this is accompanied by a faster release of EVs. Conversely, hydrogels with smaller pore sizes yield the opposite result. Currently, there are many kinds of biomaterials that can be cross-linked to create hydrogels, such as chitosan [44], collagen [45], sodium alginate, gelatin methacryloyl (GelMA) [46,47], polyethylene glycol (PEG), pluronic^®^F-127, hyaluronic acid (HA) [48], and other natural and synthetic biomaterials [49]. Natural hydrogels typically exhibit superior biocompatibility but often have poor mechanical properties. In contrast, greater customization potentials and improved mechanical strengths can be obtained from synthetic hydrogels; however, it is necessary to enhance their biocompatibility and biological activity in future research [41].

**Figure 2:**
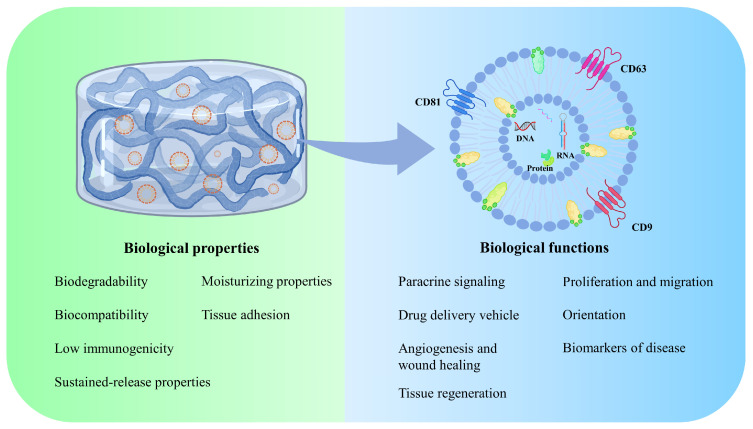
The biological properties and functions of EV-loaded hydrogels.

In this paper, we will review a combination of EVs from different sources and different types of hydrogels in the treatment of acute and chronic injuries. We will also discuss the advantages and disadvantages of these advanced therapies, with the aim of providing a valuable reference for future wound repair. Different wound healing strategies utilizing EV-loaded hydrogels have been designed and reported, making it a growing research focus. Further, the possible underlying mechanisms and signaling pathways have also been discussed. The benefits and advantages of EV-loaded hydrogels in wound healing are summarized in Figure 2.

## 5. EV-Loaded Hydrogels for Wound Healing Therapies

EV-loaded hydrogels have demonstrated the ability to promote wound repair to some extent. Although the specific signaling pathways are not fully verified at present, EVs from different cell sources play a role in regulating the signal transduction of cells around the wound. This is mainly manifested in promoting the inflammatory response at the wound site, the proliferation and migration of fibroblasts, microvascular regeneration, and the formation of epithelial tissue in other stages of healing [50]. Indeed, there have been numerous explorations involving the combination of EVs from different sources with hydrogels for wound healing.

### 5.1. Stem Cell-Derived EV-Loaded Hydrogels

At present, among various cell-derived EVs, umbilical cord mesenchymal stem cell-derived EVs are widely explored. Human umbilical cord-derived mesenchymal stem cells (hUC-MSCs) are multifunctional stem cells found in neonatal umbilical cord tissue. hUC-MSCs possess advantages such as high cellular origin, high differentiation potential, and low immunogenicity [51] and are easier to isolate compared to bone marrow-derived stem cells. In particular, hUC-MSCs have unique advantages in regenerative medicine as a promising strategy for repairing skin damage. However, due to the immunogenicity of cell therapy, the application of EVs from hUC-MSCs is obviously attractive. hUC-MSC-derived EVs have been used in combination with different hydrogel materials, such as chitosan, GelMA, F127, etc., for the treatment of diabetic wounds. It has been reported that hUC-MSC-derived EV-loaded hydrogels promote cell proliferation and migration, thereby accelerating the wound healing process [52]. The likely explanation is that the wound healing process is positively associated with levels of VEGF and TGFβ-1 expression. A study also found that hUC-MSC-derived EVs activate the Wnt/β-catenin signaling pathway to up-regulate the expression of downstream protein targets N-cadherin, cyclin-D1, and cyclin-D3, thereby stimulating the proliferation and migration of fibroblasts and keratinocytes, promoting the production of collagen and fibronectin, and leading to faster wound healing [52,53].

At the same time, the role of EVs derived from bone marrow mesenchymal stem cells (BMSCs) in regenerative medicine is crucial. Studies have reported that BMSC-derived EVs can promote wound healing by enhancing the proliferation and migration of epithelial cells and inducing angiogenesis. More importantly, EVs loaded onto hydrogels have shown superior tissue repair ability and regeneration effects [54,55,56]. Bone marrow mesenchymal stem cell-derived EVs contain various types of DNA, RNA, growth factors, and other kinds of proteins, which can be transferred to target cells and mediate cell signaling, providing a new strategy for wound healing. Research indicates that BMSC-derived EVs not only promoted angiogenesis but also enhanced the transformation of M1-type macrophages to the anti-inflammatory M2 type, thus reducing inflammation. The underlying mechanism may involve BMSC-derived EVs that promote angiogenesis by activating the VEGF signaling pathway [56]. The antioxidant abilities of miR-4645-5p and MSC-EVs within hydrogel may synergistically inhibit the SREBP2 activity of macrophages and promote the transformation of M2 macrophages [57]. Hydrogel scaffolds also provide physical protection of the wound surface and enhance cell adhesion around the wound tissue [58]. More importantly, the addition of hydrogel can sustain the release of EVs from the wound, better stimulating wound healing [54].

Growth factor therapy is one of the promising therapies that promotes cell proliferation and wound healing in skin injury [59]. VEGF is conducive to angiogenesis and wound healing [60], and the obstruction of angiogenesis is a vital cause of the difficult healing of diabetic wounds. Adipose-derived stem cell-derived EVs (ADSC-EVs) promote wound healing by regulating oxidative stress, which is related to the expression of heat shock protein 90 (HSP90) on the surface of EVs and activates the downstream eHSP90/LRP1/AKT pathway, thereby promoting the proliferation and migration of keratinocytes, fibroblasts, and endothelial cells. These cellular activities ultimately work together to promote wound healing [61,62]. Similarly, it has been shown that hypoxia-treated ADSCs-EVs can promote the proliferation and migration of fibroblasts through the PI3K/AKT pathway, thereby enhancing the secretion of VEGF and ECM, promoting diabetic wound healing, and inhibiting inflammation [31]. Other studies have reported that ADSCs-EVs containing let-7b-5p and miR-24-3p loaded onto hydrogel could significantly mediate the transition of macrophages to the CD301b overexpression phenotype [63]. Pluronic-F127 is a commonly used polymer hydrogel matrix with temperature-sensitive properties. A PF-127/hADSCs-Exos composite hydrogel rapidly covered the wound surface, and experimental results demonstrated its effectiveness in promoting skin wound healing. This composite hydrogel up-regulated the expression of Ki67, α-smooth muscle actin (α-SMA), and CD31, thereby promoting the reepithelialization of the defect site [64]. In another study, VH298 EVs were incorporated into a GelMA hydrogel, exhibiting the proliferative activity of human umbilical vein endothelial cells through the activation of the HIF-1α pathway. This contribution ultimately facilitated vascular regeneration and wound healing in vivo [65]. For scarless wound healing, exosomes derived from induced pluripotent stem cells–mesenchymal stem cells (iPSC-MSCs) were loaded onto temperature-sensitive chitosan hydrogels, which was found to promote corneal epithelial and stromal regeneration and reduce scar formation in vivo by regulating miR-432-5p and its target gene TRAM2 [66].

### 5.2. Platelet-Derived EV-Loaded Hydrogels

Recently, platelet-derived EVs (PEV) have gained attention from researchers in the field of wound healing. As reported in previous studies [67], the gelatin–alginate saline gel of graphene oxide (RGO), with the innovative addition of PEV, can regulate the immune response in diabetic wounds. This innovative hydrogel formulation was found to promote the transformation of macrophages from the pro-inflammatory M1 phenotype to the pro-healing M2 phenotype and eliminate reactive oxygen species (ROS) at the wound site. More interestingly, this hydrogel increased the expression of heat shock proteins involved in cytoprotective pathways. Similarly, platelet-derived EVs (PRP-EXOs) and zedoary turmeric homogeneous polysaccharides (ZWPs), either alone or in combination, were shown to enhance the wound healing rate. In particular, the combination of PRP-EXOs/ZWPs was more successful. This seems to be related to the up-regulation of collagen synthesis and deposition in the wound and the stimulation of angiogenesis, thereby accelerating the healing of diabetic wounds [68]. Improving the control of inflammatory responses and reducing oxidative stress in the wound microenvironment can enhance the absorption of proliferative drugs on the wound surface. In addition, studies have shown that GelMA/SFMA composite hydrogels can induce the sustained release of EVs and resveratrol at the wound site. This leads to a reduction in the expression of tumor necrosis factor-α (TNF-α) and inducible nitric oxide synthase (iNOS) while increasing the expression of anti-inflammatory factors such as transforming growth factor-β (TGF-β) and arginase-1 (Arg-1) [69]. Interestingly, this regulatory mechanism seems to be related to the enhanced expression of CD73 and adenosine receptor 2A (A2A-R) in the composite hydrogel.

### 5.3. Human Umbilical Vein Endothelial Cell-Derived EV-Loaded Hydrogels

Human umbilical vein endothelial cell-derived EVs (HUVEC-EVs) have been shown to enhance the healing of diabetic wounds by directly regulating the proliferation and migration of vascular endothelial cells and fibroblasts. Additionally, it has been demonstrated that nuclear transcription factor 2 (NRF2, a key player in anti-oxidative stress) and transcriptional activator 3 (ATF3, which can attenuate the inflammatory response) are potential targets of EVs [70], facilitating the regulation of ROS and inflammation. In addition, it has been revealed that different stressors can alter the composition and function of EVs. When HUVECs were cultured in hypoxic conditions, the secreted EVs contained an abundance of lncHAR1B, which interacted with BHLHE23 to modulate the KLF4 transcription factor, thereby promoting M1 to M2 macrophage polarization. Notably, HTGM-QCS hydrogels loaded with the hypoxic-treated HUVEC-EVs resulted in accelerated wound healing compared to other controls [71]. Furthermore, HUVEC-EVs were shown to significantly up-regulate the expression of anti-inflammatory cytokines (IL-10) while down-regulating pro-inflammatory cytokines (IL-1β and IL-6) in vivo, potentially through inducing the M2 polarization of macrophages via the DDX3X/NLRP3 regulatory axis [72]. Alleviating inflammation is significant in diabetic wound repair, which advances healing progression and avoids irritated damage to surrounding tissue. A novel biological wound dressing of a GelMA/PEGDA hydrogel coated with EVs combined with the anti-inflammatory drug tazarotene achieved the sustained release of tazarotene and EVs for 8 and 10 days, respectively, in vitro, demonstrating significant angiogenic potential to treat diabetic skin wounds [73].

### 5.4. Other Cell-Derived EV-Loaded Hydrogels

Previous studies have found that the high expression of CD301b promotes the expression of insulin-like growth factor-1 and platelet-derived growth factor-C, thereby promoting the proliferation and migration of fibroblasts [74]. At the same time, EVs derived from fibroblasts have also been shown to promote wound healing by promoting the proliferation, migration, and scar-free healing-related gene expression of fibroblasts in vitro. L929-EV treatment also promoted endothelial cell migration and tube formation. The combined application of L929-EVs and fibrin could promote collagen formation, collagen maturation, and the angiogenesis of skin wounds in mice, thereby promoting wound healing. The role of fibroblast-derived EVs in wound healing may be an important phenomenon, and such fibroblast-derived EVs could be exploited for wound healing therapy [12]. Similarly, EVs themselves act as drug delivery carriers, and combining EVs with hydrogels can enhance the drug’s ability to exert its therapeutic effect. In fact, M2-EVs implement anti-inflammatory effects by promoting the polarization of the M2 phenotype, mainly through homing and paracrine mechanisms. The hydrogel system contains chlorhexidine (CHX), which helps inhibit microbial growth and biofilm formation, resulting in 75% wound closure within 7 days of treatment. Persistent inflammation at the wound site inhibits cell proliferation and migration, and impaired angiogenesis is an important reason for the difficulty in diabetic wound healing [75,76].

### 5.5. New Strategies for EV-Loaded Hydrogels

In recent years, research on engineered EVs and bionic nanovesicles (NVs) derived from bacteria and plants has become increasingly popular for wound healing. These new vesicles have physical, chemical, and biological characteristics similar to those of EVs from animal sources [77,78].

NVs with stable expression of TNF-R1 were engineered using genetic bioengineering technology to antagonize the TNF-α-mediated pro-inflammatory signal and inhibit the NF-κB signaling pathway, thereby reducing the inflammatory response [79]. Specific miRNAs can regulate the expression level of TNF-α, and lentiviral transfection into cells produces engineered EVs containing 16 times more miR-17-5p than the original cells. Targeting p21 and PTEN through the Wnt/β-catenin signaling pathway inhibits cell aging and increases Nrf2 expression for antioxidant stress, thereby obtaining protective effects from excess ROS. The regulation of VEGFA and VEGFR2 affects endothelial cell function and may be a selective strategy to regulate vascular remodeling [80].

In fact, EVs contain a variety of signaling proteins and cytokines. These components can regulate the cell population around the wound, prompting them to respond through various signaling mechanisms. As a result, EVs play a pleiotropic role in wound healing. Table 1 summarizes some studies on EV-loaded hydrogels in promoting wound healing in recent years and how they play a role in wound healing, as well as the relevant cellular metabolisms and signaling pathways that may be involved.

All the studies summarized above show that hydrogels encapsulating EVs are capable of promoting wound healing. Various cellular metabolism levels and molecular pathways are involved, which may depend on the different sources that EVs are isolated from.

## 6. Limitations and Challenges

As mentioned above, EVs combined with hydrogel biomaterials have shown great potential in wound healing, and hydrogel encapsulates EVs in it, which greatly reduces the removal of EVs to achieve a sustained release effect and prolongs the retention of EVs at the wound site, thereby improving the treatment efficiency [88]. At the same time, the degradation products of hydrogels can also act as “sacrificial substrates” around the wound, delaying the erosion of wound tissue by a variety of enzymes at the wound, which is beneficial for treating some chronic wounds.

However, several challenges remain before the translation of EV-loaded hydrogels into clinical applications can be achieved. The first challenge is related to the extraction and preservation of EVs. The extraction of native EVs requires that the integrity of the vesicles and the activity of special proteins inside and outside the membrane be preserved as much as possible. A variety of methods have been developed to isolate EVs from body fluids [89] to achieve high recovery, high purity, and high throughput separation. The most commonly used and classical method is ultracentrifugation [90]. This method can be further divided into two techniques: ultracentrifugation and gradient density ultracentrifugation. However, prolonged ultracentrifugation may disrupt the structural and biological integrity of exosomes, causing aggregation and leading to the co-segregation of contaminating non-exosomal particles and proteins [91]. The sucrose density gradient can then be used to separate vesicle types based on density and further purify vesicles from protein aggregates [92]. The second is the immunoaffinity separation method for the selective capture of sub-populational EVs with surface-specific marker proteins, which is based on the antigen–antibody reaction to captured EVs [93]. In addition, ultrafiltration is a membrane separation technology that works based on the size and molecular weight of EVs and the separation principle of other contents, and it is usually used in combination with ultracentrifugation to reduce processing times and simplify operations. There are also polymer-based precipitation separation methods that bind water molecules and precipitate EVs by exploiting the hydrophobicity of EV membranes and other water-soluble compounds. Moreover, there are also antibody-modified magnetic bead separation methods, acoustic liquid separation methods [94], and dielectric electrophoresis separation methods [95,96]. However, there is currently a dearth of standardized methods and procedures for the isolation of EVs in existing research. This variability in separation techniques can impact the molecular weight and functionality of EVs, which is a critical factor to be analyzed before their clinical application. It is essential to prepare EVs that meet the specific requirements of clinical practice. So far, various extraction methods are still in the stages of continuous development to achieve better contents, purities, and biological activities of EVs.

More interestingly, in recent years, some studies have begun to prepare bionic EVs in order to prepare nano-sized bionic EVs faster and more efficiently. Such vesicles are more malleable and have low immunogenicity compared to natural EVs [97,98]. On the other hand, the preservation of EVs also comes with specific requirements. Repeated freezing and thawing can lead to the loss of membrane structure and protein degradation. Previous studies have shown that the addition of hydrogels can limit the Brownian motion of particles and achieve the long-term preservation of EVs by reducing the collision cracking of particles [99]. In addition, the development of hydrogels with antimicrobial properties should be an important issue in the future since some wound healing processes involve the presence of bacteria [100].

EV-loaded hydrogels still present other challenges before clinical translation. For example, it is still unclear whether the gel phase transition leads to the rupture of EVs within the internal 3D network structure. Furthermore, it is significant to consider that the crosslinking process of certain photo-crosslinked or temperature-sensitive hydrogels may lead to a decrease in the activity of sensitive gene cargoes (such as miRNA and mRNAs) [101,102]. Additionally, it is unknown if the time of in situ gel phase transition can be controlled. Moreover, the temperature and pH value of hydrogels and EVs are not uniform, and changes in storage conditions may affect the activity of EVs. Previous studies have found that the storage of EVs at −80 °C is relatively favorable for preserving the protein activity [103]. However, changes in temperature and pH value in the freeze–thaw process result in the degradation of EVs, impacting subsequent experiments on animals and potentially humans. Will EVs be deactivated by body temperature? The corresponding solutions need to be investigated in follow-up research.

In the actual clinical treatment process, there are many types of wounds, not only limited to acute wounds on the skin surface but also burns, scalds, crush injuries, etc. The treatment of chronic wounds is intractable and complicated clinically, among which, diabetic wounds are the most common. At present, the laboratory study of chronic wound models is mainly based on diabetic ulcer models, but other types of chronic wounds are intrinsically different from diabetic ulcers, and their disease models are far from developed. It is highly important to design and create various animal models to reflect actual clinical situations more accurately. Different types of wounds require hydrogels with varying performance characteristics, necessitating adjustments in the raw materials used to prepare the hydrogels. At the same time, how to modulate the stiffness, viscosity, and modulus of hydrogels to meet the pathological characteristics of different wound tissues is also one of the challenges to be overcome.

## 7. Conclusions and Prospects

As a novel wound healing treatment, EV-coated hydrogels modulate dynamic interactions and significantly enhance wound healing in both acute and chronic wounds. More importantly, the intervention of hydrogel can achieve the sustained release of EVs, realizing a longer retainment in the damaged tissue and providing a long-lasting therapeutic effect. However, it is worth noting that there are still challenges and opportunities in the subsequent clinical translation process. Although good research results have been achieved, the related molecular mechanisms of EVs in tissue repair still need to be further explored. Moreover, it is necessary to introduce the relevant industry gold standards for the isolation and preservation of EVs. Due to the modifiability and versatility of the surface molecules of engineered EVs, engineered EV–hydrogel systems may become a promising research focus. The cells can be pre-treated with drugs or modified by genetic engineering to induce the specific secretion of engineered EVs to match the pathophysiological characteristics of different wound tissues. Drugs or compounds can also be directly modified with EVs to enhance their regenerative potential. Also, 3D printing technology can be used to customize hydrogels with specific porosities and geometric shapes, providing new possibilities for the delivery of EVs. For the regenerative needs of different tissues (such as the cornea, cartilage, nerves, etc.), EV-loaded hydrogels with specific functions can be designed to achieve tissue-specific repair and regeneration. Furthermore, the immunogenicity of EVs must be taken into consideration, given that the EVs currently under study may contain animal-derived substances. With the continuous development of biomaterials science, EV-loaded hydrogels will achieve new breakthroughs in the field of tissue engineering and regenerative medicine.

## Figures and Tables

**Table 1:** Effect of extracellular vesicles from different sources on wound healing.

EV Source		Biomaterial	Wound Model	Experimental Results or Underlying Mechanism	Reference
Umbilical cord	MSC	Chitosan	Diabetic wound	Promote cell migration and angiogenesis	[81]
		Genipin	Diabetic wound	Accelerate cell proliferation and migration New collagen deposition Reduce inflammation	[82]
		SC	Diabetic wound	Pro-angiogenesis Activation of the HIF-1α/VEGF pathway	[83]
		GelMA	Diabetic wound	Promote the proliferation and migration of fibroblasts Promote collagen deposition in the wound	[62]
		rhCOL III	Diabetic wound	Inhibition of inflammatory responses Promote cell proliferation and angiogenesis CD31↑; α-SMA↑ Ki67↑; IL6↓	[45]
		F127	Diabetic wound	CD31↑; Ki67↑ Granulation tissue regeneration↑ VEGF↑; TGFβ-1↑	[52]
Umbilical veins	HUVEC	MC	Diabetic wound	Increase angiogenesis Muscle protection Activation of VEGF/VEGFR pathway and autophagy-lysosomal pathway	[84]
		GelMA/PEGDA microneedles	Diabetic wound	Angiogenesis↑ Collagen deposition↑ Cell migration↑ CD31↑; α-SMA↑	[73]
Bone marrow	MSC	CEC DCMC	Diabetic wound	Self-healing properties and mechanical stability Stimulate angiogenesis Promote the transformation of M1 macrophages into M2 macrophages VEGF signaling pathway	[56]
		NAGA/GelMA/Lapite/glycerin	Acute wound	Cell proliferation↑ Tissue formation, remodeling, and re-epithelialization	[54]
		GelMA-dopamine	Diabetic wound	Cell migration↑ Angiogenesis↑ Collagen deposition↑ Regeneration of skin appendages↑ IL-6↑ CD31↑ TGF-β↑	[55]
Adipose	MSC	F127	Acute wound	Ki67↑ α-SMA↑ CD31↑ Skin barrier proteins (KRT1, AQP3) ↑	[64]
		PVA/GA	Acute wound	CD31↑ F4/80↑ CD86↓ miR-192-5p miR-29a Hypertrophic scars↓	[85]
		HA	Acute wound	CD301b ↑ Proliferation of fibroblasts↑ Regulation of let-7b-5p, miR-24-3p, and other miRNAs in the change of macrophages to the CD301bhi phenotype Collagen↑	[63]
Platelets		GelMA SFMA	Diabetic wound	iNOS ↓ Regulate the phenotypic transformation of macrophages TNF-α ↓ Angiogenesis ↑	[69]
		RGO GelAlg	Diabetic wound	Inflammatory biomarkers↓ Heat shock proteins involved in cellular protective pathways ↑ Angiogenesis↑ Hair follicle regeneration	[67]
		Chitosan/silk	Diabetic wound	Wound re-epithelialization↑ Collagen synthesis Skin angiogenesis	[68]
Macrophage	M2	HA@MnO_2_	Diabetic wound	Antibacterial Catalytic H_2_O_2_ Antioxidant properties Angiogenesis Collagen deposition	[59]
		PEG	Acute wound	Macrophage M1 → M2 iNOS↓, CD206↑, ARG1↑	[38]
Fibroblast		Fibrin glue	Acute wound	Cell migration Tube formation VEFG↑, CD31↑, β-catenin↑ Deposition and maturation of collagen protein	[12]
Polymorphonuclear neutrophils	PMN	ECM	Diabetic wound	Antibacterial Angiogenesis	[40]
Royal jelly		Serma	Acute wound	Cell proliferation Angiogenesis IL-10/TGF-β1 ↑ TNF-α/IL-6↓	[86]
Placental	MSC	MC-CS	Diabetic wound	Bcl-2/Bax/VEGF↑ Cell proliferation ECM generation↑	[87]
Human umbilical cord blood (HUCB)	MNC	HA-PCL	Diabetic wound	miRNAs (150-5p, 181a-5p, let-7a-5p, 342-3p, let-7f-5p, 2233p, 142-3p) ↑ Proliferation of epidermal keratinocytes and ECs	[48]
Epidermal	ESCs	GelMA	Diabetic wound	Angiogenesis Cell migration HIF-1 α/VEGF-A signaling pathway	[65]

EVs: extracellular vesicles; ↑: increase; ↓: decrease; MC: methyl cellulose; CS: chitosan; SC: small intestine submucosa; F127: pluronic-F127; PVA: polyvinyl alcohol; ECM: extracellular matrix; VEGF: vascular endothelial growth factor; TGFβ-1: transforming growth factor β-1; CEC: carboxyethyl chitosan; DCMC: dialdehyde carboxymethyl cellulose; RGO: graphene oxide; GelAlg: alginate gelatin; Serma: methacrylated silk fibroin protein.

## Abbreviations

**Abbreviation**	**Full Name**
EVs	extracellular vesicles
ECM	extracellular matrix
MMPs	matrix metalloproteinases
VEGF	vascular endothelial growth factor
GelMA	gelatin methacryloyl
HA	hyaluronic acid
F127	pluronic F-127
hUC-MSCs	human umbilical cord-derived mesenchymal stem cells
BMSCs	bone marrow mesenchymal stem cells
ADSCs	adipose-derived stem cells
HSP90	heat shock protein 90
α-SMA	α-smooth muscle actin
iPSC-MSCs	induced pluripotent stem cells–mesenchymal stem cells
PEVs	platelet-derived EVs
RGO	graphene oxide
ROS	oxygen species
ZWP	zedoary turmeric homogeneous polysaccharide
TNF-α	tumor necrosis factor-α
iNOS	inducible nitric oxide synthase
TGF-β	transforming growth factor-β
Arg-1	arginase-1
A2A-R	adenosine receptor 2A
HUVEC-EVs	human umbilical vein endothelial cell-derived EVs
IL	interleukin
NRF2	nuclear transcription factor 2
ATF3	transcriptional activator 3
NVs	nanovesicles
